# Association between mean arterial pressure during the first 24 hours and hospital mortality in patients with cardiogenic shock

**DOI:** 10.1186/s13054-020-03217-6

**Published:** 2020-08-20

**Authors:** Barry Burstein, Meir Tabi, Gregory W. Barsness, Malcolm R. Bell, Kianoush Kashani, Jacob C. Jentzer

**Affiliations:** 1 Division of Pulmonary and Critical Care Medicine, Mayo Clinic, Rochester, MN USA; 2 Department of Cardiovascular Medicine, Mayo Clinic, Rochester, MN USA; 3 Division of Nephrology and Hypertension, Mayo Clinic, Rochester, MN USA; 4 Department of Internal Medicine, Mayo Clinic, 200 First Street SW, Rochester, MN 55905 USA

**Keywords:** Cardiogenic shock, Shock, Cardiac intensive care unit, Critical care, Mortality, Hypotension, Blood pressure

## Abstract

**Background:**

The optimal MAP target for patients with cardiogenic shock (CS) remains unknown. We sought to determine the relationship between mean arterial pressure (MAP) and mortality in the cardiac intensive care unit (CICU) patients with CS.

**Methods:**

Using a single-center database of CICU patients admitted between 2007 and 2015, we identified patients with an admission diagnosis of CS. MAP was measured every 15 min, and the mean of all MAP values during the first 24 h (mMAP_24_) was recorded. Multivariable logistic regression determined the relationship between mMAP_24_ and adjusted hospital mortality.

**Results:**

We included 1002 patients with a mean age of 68 ± 13.7 years, including 36% females. Admission diagnoses included acute coronary syndrome in 60%, heart failure in 74%, and cardiac arrest in 38%. Vasoactive drugs were used in 72%. The mMAP_24_ was higher (75 vs. 71 mmHg, *p* < 0.001) among hospital survivors (66%) compared with non-survivors (34%). Hospital mortality was inversely associated with mMAP_24_ (adjusted OR 0.9 per 5 mmHg higher mMAP_24_, *p* = 0.01), with a stepwise increase in hospital mortality at lower mMAP_24_. Patients with mMAP_24_ < 65 mmHg were at higher risk of hospital mortality (57% vs. 28%, adjusted OR 2.0, 95% CI 1.4–3.0, *p* < 0.001); no differences were observed between patients with mMAP_24_ 65–74 vs. ≥ 75 mmHg (*p* > 0.1).

**Conclusion:**

In patients with CS, we observed an inverse relationship between mMAP_24_ and hospital mortality. The poor outcomes in patients with mMAP_24_ < 65 mmHg provide indirect evidence supporting a MAP goal of 65 mmHg for patients with CS.

## Background

Cardiogenic shock (CS) is the second-most common form of circulatory shock in all critical care units and the most common form of shock among patients admitted to cardiac intensive care units (CICUs) [1, 2]. CS manifests in clinical, hemodynamic, and biochemical derangements characterized by arterial hypotension and tissue hypoperfusion, resulting in significant morbidity and mortality despite appropriate treatment [3]. The mainstay of management is early intervention to address the inciting cause, in conjunction with supportive care, to restore end-organ perfusion and prevent multi-organ failure and death [3]. In cases of acute myocardial infarction, emergency revascularization is indicated in order to improve cardiac function [4].

Immediate restoration of adequate systemic blood pressure using intravenous inotropes, vasopressors, and/or mechanical circulatory support is a priority in CS [3]. The optimal blood pressure target in CS must balance the maintenance of adequate end-organ perfusion with the adverse effects of excessive cardiac afterload and arrhythmias induced by catecholamine vasopressors. The ideal target mean arterial pressure (MAP) for patients with CS is unclear, and current strategies are based on evidence from patients with vasodilatory shock and cardiac arrest (CA) [3, 5, 6]. Furthermore, it has been suggested that patients with pre-existing hypertension may benefit from higher MAP goals [7], and a history of hypertension is common among patients with cardiovascular disease [8]. By contrast, recent evidence has demonstrated favorable outcomes among older patients supported with permissive hypotension (MAP 60–65 mmHg) [9].

Given the sparsity of evidence to support specific MAP targets in patients with CS, we sought to describe the relationship between MAP and hospital mortality among patients with CS. We hypothesized that hospital mortality among patients with CS would increase as a function of lower MAP and that a threshold MAP may identify an optimal MAP range. Our secondary aim was to evaluate the prevalence of organ failure as a function of MAP.

## Methods

### Study population

This study was approved by the Institutional Review Board of Mayo Clinic (IRB # 16-000722) as posing minimal risk to patients and was performed under a waiver of informed consent. We retrospectively analyzed a database containing data from the initial CICU admission for consecutive unique adult patients aged ≥ 18 years admitted to the CICU at Mayo Clinic Hospital St. Mary’s Campus between January 1, 2007, and December 31, 2015 [10–12]. The Mayo Clinic CICU is a closed unit serving critically ill cardiac medical patients, but not postoperative cardiac surgery patients and patients receiving extracorporeal membrane oxygenation (ECMO) support. We included only those patients with an admission diagnosis of CS, defined as an International Classification of Diseases (ICD)-9 code of 785.51 documented within 1 day of CICU admission. We excluded all patients without an admission diagnosis of CS (including those without available admission diagnosis data), even if they had an ICD-9 code for CS documented at another time during hospitalization. Patients without available data on MAP were also excluded. Patients who declined Minnesota Research Authorization, according to Minnesota state law statute 144.295, were excluded from the study.

### Data sources

We recorded demographic, vital sign, laboratory, clinical, and outcome data, as well as procedures and therapies performed during the CICU and hospital stay, as previously described [10–12]. All relevant data were extracted electronically from the medical record using the Mayo Clinic Multidisciplinary Epidemiology and Translational Research in Intensive Care Data Mart [13]. The admission value of all vital signs, clinical measurements, and laboratory values was defined as either the first value recorded after CICU admission or the value recorded closest to CICU admission. In addition, vital signs were recorded every 15 min, and the maximum, minimum, and mean values over the first 1, 6, and 24 h were recorded. Blood pressure was preferentially recorded from invasive measurements, when available, and otherwise was recorded from noninvasive measurements. Peak vasopressor and inotrope doses were used to calculate the Vasoactive-Inotropic Score [14]. Admission diagnoses included all ICD-9 diagnostic codes recorded on the day of CICU admission and the day before or after CICU admission; these admission diagnoses were not mutually exclusive, and the primary admission diagnosis could not be determined. Admission diagnoses of interest included CS, acute coronary syndrome (ACS), heart failure (HF), supraventricular tachycardia, atrial fibrillation, ventricular fibrillation, ventricular tachycardia, CA, respiratory failure, and sepsis. Discharge ICD-9 diagnostic codes were reviewed for a diagnosis of hypertension. Severe acute kidney injury (AKI) was defined as KDIGO stage 2 or 3 AKI during the CICU stay (i.e., doubling of serum creatinine or increase in serum creatinine to ≥ 4.0 mg/dl or new dialysis initiation in the CICU); mild AKI was defined as KDIGO stage 1 AKI (an increase in creatinine by ≥ 0.3 mg/dl or 50% from baseline) [11, 15]. Baseline creatinine was considered to be the latest creatinine within 1 year prior to the index hospital admission, and patients who had previously received dialysis were excluded from this AKI analysis. Non-cardiovascular organ failure was defined as a score ≥ 3 on any day 1 SOFA organ subscore [16].

### Statistical analysis

The primary endpoint was all-cause hospital mortality; secondary endpoints included CICU mortality and post-discharge mortality up to 1 year among hospital survivors. Mortality and other outcome data were extracted from Mayo Clinic electronic databases, the state of Minnesota electronic death certificates, and the Rochester Epidemiology Project database [17]. Categorical variables are reported as number (percentage), and the Pearson chi-squared test was used to compare groups. Continuous variables are reported as mean (± standard deviation); the Wilcoxon rank-sum test was used to compare groups. The calculation of 24-h mean MAP (mMAP_24_) was performed using invasive blood pressure measurements, if available; otherwise, mMAP_24_ was calculated using noninvasive blood pressure values. Logistic regression was used to determine the association between mMAP_24_ with hospital mortality before and after adjusting for age, gender, race, Charlson Comorbidity Index (CCI), and Acute Physiology and Chronic Health Evaluation IV (APACHE-IV) predicted mortality; admission diagnoses of CA, sepsis, HF, and ACS; peak 24-h VIS; and the use of intra-aortic balloon pump (IABP), dialysis, pulmonary artery catheter (PAC), coronary angiography, percutaneous coronary intervention (PCI), and mechanical ventilation. Subgroup analysis was performed by repeating multivariable logistic regression after excluding patients with SCAI shock stages A or B or sepsis; besides, logistic regression was repeated in the overall cohort after adjusting for SCAI shock stages. Discrimination was assessed using the area under the receiver-operator characteristic curve (AUC, *c*-statistic) value, and the optimal cutoff defined using Youden’s *J* index. Post-discharge survival among hospital survivors was evaluated using the Kaplan-Meier survival analysis and Cox proportional-hazards analysis. Two-tailed *p* values < 0.05 were considered statistically significant. Statistical analyses were performed using JMP Pro version 14.1.0 (SAS Institute, Cary, NC).

## Results

### Study population

The database included 10,004 unique CICU patient admissions, of whom 1078 had an admission diagnosis CS and were potentially eligible for inclusion [10]. We excluded 76 of these patients due to lack of available data for MAP (Supplemental Figure 1). The final study population of 1002 unique patients had a mean age of 67.7 ± 13.7 years, including 36.4% females (Table 1). The mean CCI was 2.4 ± 2.5, and the mean APACHE-IV predicted hospital mortality was 38.4% ± 29.3 overall. Concomitant admission diagnoses included ACS in 599 (59.8%) patients, HF in 740 (73.9%), sepsis in 199 (19.9%), and CA in 379 (37.8%); 77 (7.7%) patients had neither ACS nor HF as an admission diagnosis. Non-cardiovascular organ failure developed on the first day in 690 (68.9%) patients; 630 (73.2%) patients developed AKI during the CICU stay, including 314 (36.5%) with severe AKI.

**Table 1:** Baseline characteristics of the study population. Data represented as mean ± standard deviation for continuous variables and *n* (%) for categorical variables

	Overall (*n* = 1002)	MAP < 65 mmHg (*n* = 186)	MAP 65–75 mmHg (*n* = 426)	MAP ≥ 75 mmHg (*n* = 390)	*p* value
*Demographics*					
Age	67.7 ± 13.8				
Female gender	365 (36.4%)	75 (40.3%)	156 (36.6%)	134 (34.4%)	0.38
White race	921 (91.9%)	173 (93.0%)	385 (90.4%)	363 (93.1%)	0.31
Body mass index	29.3 ± 6.8	29.9 ± 8.1	29.1 ± 6.4	29.5 ± 6.6	0.59
*Severity of illness*					
APACHE-III score	86.2 ± 33.0	100.8 ± 35.3	87.5 ± 32.0	77.9 ± 30.2	< 0.0001
APACHE-IV predicted mortality	0.38 ± 0.29	0.48 ± 0.29	0.40 ± 0.29	0.32 ± 0.28	< 0.0001
SOFA score	7.6 ± 4.2	9.5 ± 4.4	7.9 ± 4.1	6.4 ± 3.8	< 0.0001
Maximum week 1 SOFA score	8.4 ± 4.2	10.0 ± 4.4	8.7 ± 4.1	7.3 ± 3.9	< 0.0001
Cardiovascular SOFA score	2.5 ± 1.3	3.0 ± 1.2	2.6 ± 1.2	2.1 ± 1.3	< 0.0001
*Comorbidities*					
Charlson Comorbidity Index	2.4 ± 2.5	2.84 ± 2.7	2.4 ± 2.5	2.1 ± 2.4	0.01
Prior myocardial infarction	200 (20.0%)	35 (18.8%)	95 (22.4%)	70 (18.1%)	0.29
Prior heart failure	193 (19.4%)	48 (25.8%)	90 (21.2%)	55 (14.3%)	0.002
Prior hypertension	348 (34.7%)	64 (34.4%)	145 (34.0%)	139 (35.6%)	0.89
Prior diabetes mellitus	287 (28.8%)	60 (32.3%)	119 (28.0%)	108 (28.0%)	0.51
Prior CKD	202 (20.2%)	49 (26.3%)	82 (19.3%)	71 (18.4%)	0.07
Prior dialysis	88 (8.8%)	32 (17.2%)	34 (8.0%)	22 (5.6%)	0.0001
Prior stroke	121 (12.1%)	23 (12.4%)	58 (13.7%)	40 (10.4%)	0.36
Prior cancer	214 (21.5%)	54 (29.0%)	97 (22.8%)	63 (16.3%)	0.002
*Admission diagnoses*					
Cardiac arrest	379 (37.8%)	63 (33.9%)	149 (35.0%)	167 (42.8%)	0.03
Sepsis	199 (19.9%)	46 (24.7%)	96 (22.5%)	57 (14.6%)	0.003
Respiratory failure	645 (64.4%)	115 (61.8%)	288 (67.6%)	242 (62.1%)	0.18
Acute coronary syndrome	599 (59.8%)	98 (52.7%)	260 (61.0%)	241 (61.8%)	0.09
Heart failure	740 (73.9%)	139 (74.7%)	326 (76.5%)	275 (70.5%)	0.14
*SCAI cardiogenic shock stage*					
Stage A	151 (15.1%)	7 (3.8%)	63 (14.8%)	81 (20.8%)	< 0.0001
Stage B	336 (33.5%)	50 (26.9%	146 (34.3%)	140 (35.9%)	< 0.0001
Stage C	116 (11.6%)	19 (10.2%)	41 (9.6%)	56 (14.4%)	< 0.0001
Stage D	329 (32.8%)	84 (45.2%)	150 (35.2%)	95 (24.4%)	< 0.0001
Stage E	70 (7.0%)	26 (14.0%)	26 (6.1%)	18 (4.6%)	< 0.0001
*CICU admission clinical parameters*					
Heart rate (HR, beats per minute)	90.7 ± 24.1	93.8 ± 26.8	91.3 ± 23.7	88.6 ± 22.9	0.09
Systolic blood pressure (SBP, mmHg)	110.8 ± 27.8	97.0 ± 24.0	105.9 ± 23.6	120.0 ± 30.6	< 0.0001
Diastolic blood pressure (mmHg)	65.3 ± 18.8	56.9 ± 15.9	63.9 ± 17.0	71.8 ± 19.7	< 0.0001
Mean arterial pressure (first 24 h, mmHg)	73.4 ± 10.1	68.5 ± 18.0	76.9 ± 17.3	87.4 ± 20.9	< 0.0001
Shock index (HR/SBP)	0.86 ± 0.30	1.0 ± 0.31	0.87 ± 0.3	0.79 ± 0.9	< 0.0001
Oxygen saturation (%)	92.9 ± 10.9	91.3 ± 12.1	92.6 ± 11.6	93.9 ± 9.2	0.006
*Admission laboratory values*					
Sodium	137.0 ± 5.1	137.1 ± 5.9	136.7 ± 5.0	137.3 ± 4.8	0.11
Potassium	4.3 ± 0.9	4.4 ± 0.9	4.4 ± 0.87	4.3 ± 0.8	0.08
Bicarbonate	21.2 ± 5.2	20.9 ± 6.0	21.0 ± 5.1	21.5 ± 4.8	0.18
Anion gap	14.1 ± 4.8	16.4 ± 6.0	13.7 ± 4.8	13.8 ± 4.0	< 0.0001
Creatinine	1.6 ± 1.1	2.2 ± 1.6	1.5 ± 1.0	1.3 ± 0.8	< 0.0001
Hemoglobin	12.1 ± 2.4	11.1 ± 2.1	12.0 ± 2.3	12.7 ± 2.4	< 0.0001
Lactate	3.9 ± 3.7	5.1 ± 4.6	3.6 ± 3.4	3.7 ± 3.4	0.009
Troponin (initial)	2.3 ± 4.5	2.7 ± 6.6	2.2 ± 4.2	2.1 ± 3.8	0.48
Troponin (peak during hospital stay)	3.9 ± 6.7	4.5 ± 8.9	3.9 ± 6.8	3.7 ± 5.4	0.57
*Procedures and therapies*					
Number of vasoactive drugs	1.5 ± 1.3	2.0 ± 1.4	1.6 ± 1.2	1.1 ± 1.3	< 0.0001
Vasopressors	722 (72.1%)	161 (86.6%)	332 (77.9%)	229 (58.7%)	< 0.0001
Inotropes	282 (28.1%)	68 (36.6%)	123 (28.9%)	91 (23.3%)	< 0.0001
Peak vasoactive infusion score (VIS)	26.1 ± 54.3	47.6 ± 79.0	23.8 ± 46.3	18.5 ± 44.7	< 0.0001
Invasive ventilation	599 (59.8%)	105 (56.5%)	269 (63.2%)	225 (57.7%)	0.17
Noninvasive ventilation	241 (24.1%)	42 (22.6%)	118 (27.7%)	81 (20.8%)	0.06
Dialysis	101 (10.1%)	27 (14.5%)	43 (10.1%)	31 (8.0%)	0.05
Intra-aortic balloon pump	389 (38.8%)	41 (22.0%)	193 (45.3%)	155 (39.7%)	< 0.0001
Pulmonary artery catheter	240 (24.0%)	40 (21.5%)	112 (26.3%)	88 (22.6%)	0.32
Coronary angiogram	647 (64.6%)	97 (52.2%)	275 (64.6)	275 (70.5%)	< 0.0001
Percutaneous coronary intervention	325 (32.4%)	48 (25.8%)	142 (33.3%)	135 (34.6%)	0.09
Impella®	8 (0.8%)	2 (1.1%)	5 (1.2%)	1 (0.3%)	0.25
ECMO	2 (0.2%)	0 (0%)	0 (0%)	2 (0.5%)	0.21
*Outcomes*					
Severe acute kidney injury during CICU stay	314 (36.5%)	63 (46.0%)	139 (36.7%)	112 (32.5%)	0.02
Severe acute kidney injury during hospital	392 (43.6%)	71 (49.3%)	172 (44.1%)	149 (40.9%)	0.22
CICU length of stay	4.3 ± 7.3	4.5 ± 14.1	4.6 ± 4.8	3.8 ± 3.8	< 0.0001
Hospital length of stay	13.1 ± 18.1	14.4 ± 25.4	13.7 ± 18.5	11.7 ± 12.5	0.02
CICU mortality	234 (23.3%)	80 (43.0%)	81 (19.0%)	73 (18.7%)	< 0.0001
Hospital mortality	338 (33.7%)	106 (57.0%)	127 (29.8%)	105 (26.9%)	< 0.0001

*APACHE* Acute Physiology And Chronic Health Evaluation, *BUN* blood urea nitrogen, *CICU* cardiac intensive care unit, *CKD* chronic kidney disease, *ECMO* extracorporeal membrane oxygenation, *IABP* intra-aortic balloon pump, *RBC* red blood cell, *SCAI* Society for Cardiovascular Angiography and Interventions, *SOFA* Sequential Organ Failure Assessment, *WBC* white blood cell

The mMAP_24_ for the population was 73.4 ± 10.1 mmHg. A total of 186 (18.6%) patients had a mMAP_24_ < 65 mmHg, and 390 (38.9%) patients had a mMAP_24_ ≥ 75 mmHg. During the first 24 h of the CICU stay, 719 (71.8%) patients received vasoactive drugs, including vasopressors in 668 (66.7%), and inotropes in 282 (28.1%), with a mean peak 24-h VIS of 26.1 ± 54.3. IABP was used during the CICU admission in 389 (38.8%) of patients.

**Fig. 1:**
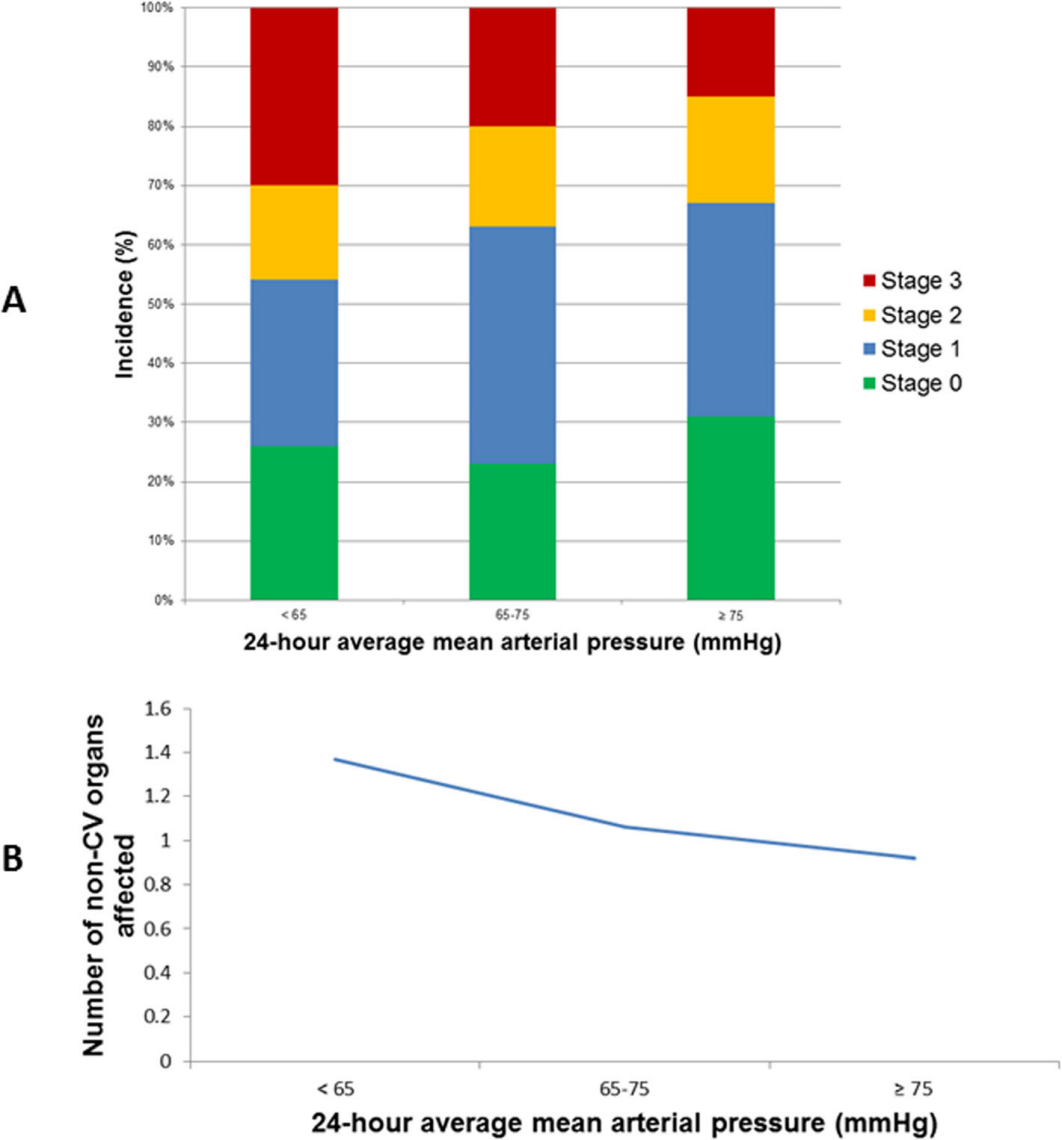
**a**, **b** Incidence of acute kidney injury by stage (**a**) and non-cardiac organ failure (by number of organs affected, **b**) as a function of the 24-h average mean arterial pressure (mMAP_24_)

Patients with a mMAP_24_ < 65 mmHg differed from patients with a mMAP_24_ 65–75 mmHg or mMAP_24_ ≥ 75 mmHg (Table 1), with greater severity of illness (APACHE-III score 100.8 ± 35.3 vs. 87.5 ± 32.0 vs. 77.9 ± 30.2, *p* < 0.001), more severe CS based on Society for Cardiovascular Angiography and Interventions (SCAI) staging, increased incidence of severe AKI (30.0% vs. 19.8% vs. 14.8%, *p* = 0.001, Fig. 1a), greater use of vasoactive infusions (85.5% vs. 78.9% vs. 54.4%, *p* < 0.001), and an increased number of non-cardiac organ injury (mean 1.4 vs. 1.1 vs. 0.9, < 0.001, Fig. 1b). Conversely, patients with mMAP_24_ < 65 mmHg underwent fewer coronary angiograms (52.5% vs. 64.6% vs. 70.5%, *p* < 0.001) and were less often supported with an IABP or Impella device (Abiomed, Danvers, MA, USA) (30.1% vs. 50.5% vs. 46.4%, respectively, *p* < 0.001).

### Hospital mortality

**Fig. 2:**
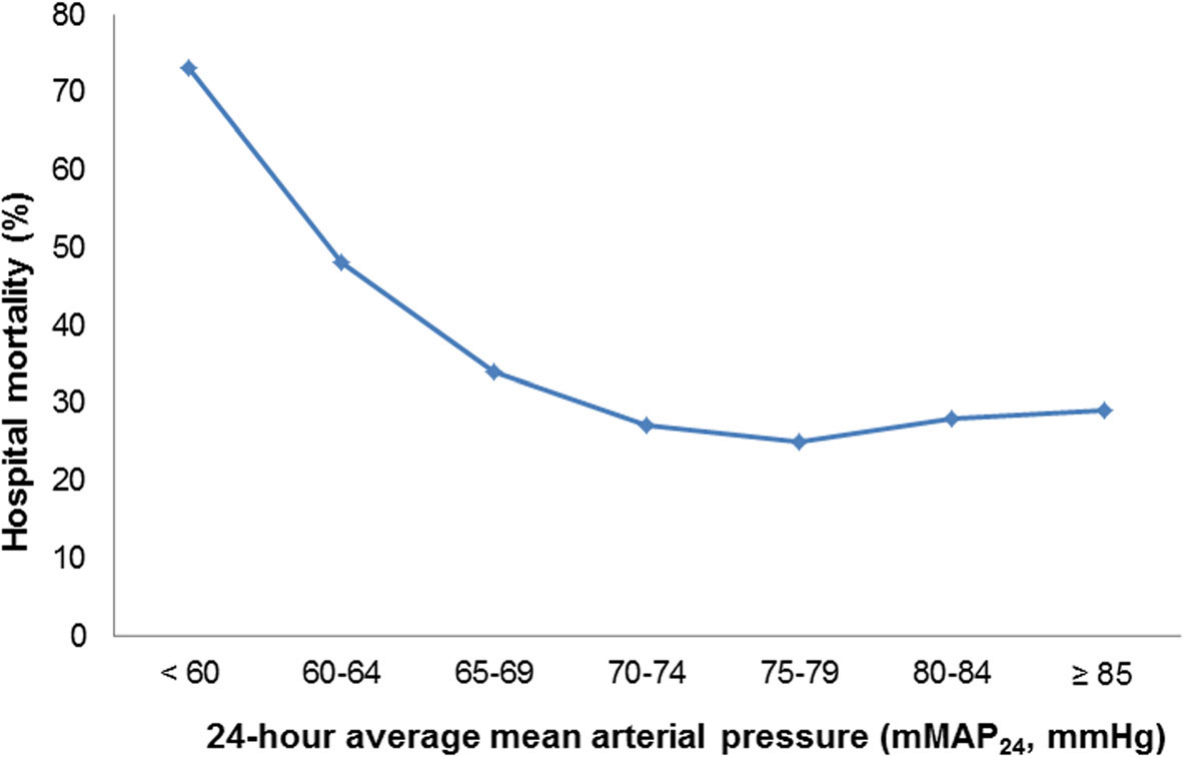
Hospital mortality as a function of the average mean arterial pressure in the first 24 h of cardiac intensive care unit (CICU) admission (mMAP_24_)

**Fig. 3:**
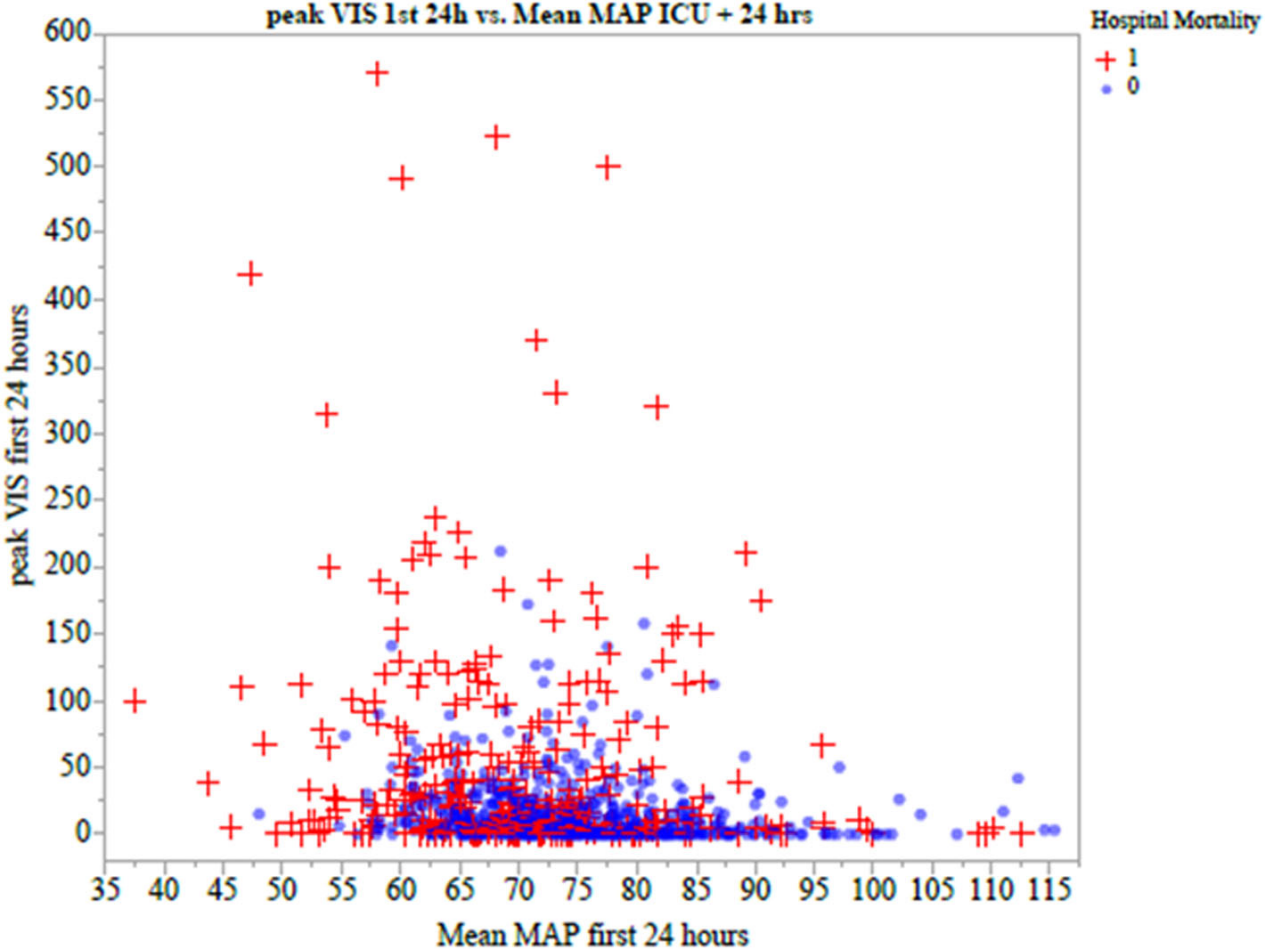
Hospital mortality as a function of 24-h average mean arterial pressure (mMAP_24_) and vasoactive infusion score (VIS)

Hospital mortality was 33.7%, including 23.4% of patients who died in the CICU. Patients who died in the hospital had lower mMAP_24_ (70.8 vs. 74.7 mmHg, *p* < 0.001). Crude hospital mortality was higher in patients with mMAP_24_ < 65 mmHg compared with patients with mMAP_24_ 65–75 mmHg or mMAP_24_ ≥ 75 mmHg (57.0% vs. 29.8% vs. 26.9%, *p* < 0.001 for mMAP_24_ < 65 mmHg vs. other groups and *p* = 0.36 between other groups). The mMAP_24_ was inversely associated with hospital mortality (unadjusted OR 0.82 per 5 mmHg higher mMAP_24_, 95% CI 0.76–0.88, *p* < 0.001; optimal cutoff 64.6 mmHg; Fig. 2). Similar findings were observed in patients with ACS or HF (Supplemental Figures 2A and 2B), patients with CA (Supplemental Figures 3A and 3B), patients with a pre-admission diagnosis of hypertension (Supplemental Figures 4A and 4B), and patients aged 65 years and older (Supplemental Figure 5). Mean values of systolic, diastolic, and mean BP were significantly lower for inpatient deaths at all time points (1, 6, and 24 h), although the magnitude of these differences was relatively modest (Supplemental Figure 6). The association between mMAP_24_ and mortality remained after excluding patients with SCAI stages A and B of CS (adjusted OR 0.850 per 5 mmHg higher, 95% CI 0.755–0.957, *p* = 0.007), as well as in the overall cohort after adjusting for SCAI CS stage (adjusted OR 0.913 per 5 mmHg, 95% CI 0.838–0.994, *p* = 0.035). The association between mMAP_24_ and hospital mortality persisted after excluding patients with an admission diagnosis of sepsis (adjusted OR 0.873, 95% CI 0.794–0.960, *p* = 0.0052). The association between mMAP_24_ and hospital mortality was present in patients without sepsis (adjusted OR 0.873, 95% CI 0.794–0.960, *p* = 0.0052). The optimal mMAP_24_ cutoff for predicting hospital mortality was 65.2 mmHg in patients with ACS and 70.0 mmHg in patients with HF. Hospital mortality varied as a function of mMAP_24_ and the maximum number of vasopressors during the first 24 h and the peak VIS during the first 24 h (Fig. 3). In subgroups of patients with and without a diagnosis of hypertension, there was no association between mMAP_24_ and the incidence of severe AKI (*p* = 0.83) (Supplemental Figures 4A and 4B).

After multivariable adjustment, mMAP_24_ remained inversely associated with hospital mortality (adjusted OR 0.89 per 5 mmHg higher mMAP_24_, 95% CI 0.82–0.97, *p* = 0.01, Table 2). Patients with a mMAP_24_ < 65 mmHg were at higher risk of hospital mortality (adjusted OR 2.05, 95% CI 1.38–3.02, *p* < 0.001), with no difference between patients with mMAP_24_ 65–75 mmHg and mMAP_24_ ≥ 75 mmHg (*p* = 0.77).

**Table 2:** Predictors of hospital mortality on multivariable regression. Only predictors with *p* < 0.1 are shown. Additional predictors with *p* ≥ 0.1 included in the model were white race, noninvasive ventilator use, creatinine, hemoglobin, PAC, RBC transfusion, respiratory failure, HF, and sepsis. Final model validation AUC 0.80

Variable	Adjusted OR	95% CI	*p* value
APACHE-IV predicted mortality	3.61	1.81–7.22	< 0.001
Cardiac arrest	3.11	2.21–4.36	< 0.001
Dialysis during CICU admission	2.64	1.68–4.14	< 0.001
mMAP_24_ < 65 mmHg	2.05	1.38–3.02	< 0.001
Charlson Comorbidity Index	1.07	1.01–1.14	0.03
Age (per year)	1.03	1.02–1.04	< 0.001
Peak vasoactive infusion score (VIS) during first 24 h	1.01	1.01–1.02	< 0.001
IABP	0.64	0.44–0.92	0.02

*Abbreviations*: *APACHE* Acute Physiology and Chronic Health Evaluation, *IABP* intra-aortic balloon pump, *mMAP*_*24*_ average mean arterial pressure (MAP) in the first 24 h

## Discussion

In this retrospective study of a large tertiary CICU patient population with CS, we demonstrate that mMAP_24_ is inversely associated with CICU and hospital mortality after adjusting for illness severity and CICU therapies, including patients with common CICU diagnoses such as ACS, HF, and CA. These data suggest that mMAP_24_ is an independent predictor of hospital mortality in CS patients across subgroups, even when accounting for vasopressor requirements. The association between mMAP_24_ and hospital mortality remained even after adjustment for the SCAI shock stage, suggesting that the importance of mMAP_24_ extends beyond initial shock severity alone. Patients who were able to maintain a MAP above 65 mmHg had lower hospital mortality; similar results were seen among patients with ACS, whereas unexpectedly patients with HF seemed to have better outcomes at a MAP above 70 mmHg. Among patients with a MAP below 65 mmHg, hospital mortality increased in proportion to the severity of hypotension, and patients with the most severe hypotension were at highest risk of mortality. We observed a threshold effect, such that patients with progressively higher mMAP_24_ above these levels did not have further decreases in mortality. The prevalence of non-cardiovascular organ failure and severe AKI was higher among patients with lower MAP, potentially explaining why these patients had higher mortality. Notably, not all patients who had an admission diagnosis of CS received vasopressors, mechanical circulatory support, or had manifest hypoperfusion on CICU admission, suggesting that some patients had resolved CS. These data suggest that maintaining MAP goals lower than 65 mmHg may not be adequate to preserve organ perfusion. However, targeting MAP goals higher than 65 mmHg may potentially expose patients to added hazards from the known adverse effects of vasoactive drugs without definite benefit. We did not observe a difference in MAP thresholds in patients with a pre-existing history of hypertension, and our findings do not suggest that a higher MAP is preferentially associated with any decrease in mortality or end-organ injury among this subgroup of patients.

Evidence-based therapies available to patients with CS remain limited, and much of the critical care strategies in the CICU have been extrapolated from other non-CS populations [3]. The optimal MAP goal in patients with CS has not been well defined. Current strategies are based on evidence from patients with other forms of circulatory shock, particularly patients with sepsis whose physiology is entirely different from CS due to the presence of a low diastolic blood pressure from vasoplegia which drives down the MAP [7, 18]. The SEPSISPAM trial compared a vasopressor strategy targeting MAP of 80–85 mmHg to a target of 65–70 mmHg in patients with septic shock and found no difference in death or AKI at 28 days despite more arrhythmias in the higher MAP arm; patients with chronic hypertension maintained at the higher MAP target were less likely to suffer from AKI [7]. By contrast, a recent multicenter randomized controlled trial of patients 65 years and older who were admitted to the ICU with septic shock demonstrated that permissive hypotension (MAP 60–65 mmHg) reduced vasopressor exposure without increasing the risk of mortality or AKI (including patients with and without hypertension) [9]. The findings of our study indirectly support the safety of a lower MAP target (i.e., 65–70 mmHg) in CS patients, but did not show a benefit of higher MAP targets among patients with a history of hypertension. Evidence supports the use of vasopressors such as norepinephrine that have a lower rate of cardiovascular adverse events, including increased myocardial oxygen demand, ischemia, arrhythmias, and mortality [1, 14]. In general, increasing doses of vasoactive agents increase the risk of cardiovascular adverse events and are associated with higher mortality, and current recommendations suggest the lowest effective dose necessary to achieve a target MAP [3, 14]. We observed a strong independent association between higher vasopressor doses based on VIS_24_ and higher hospital mortality.

Many CS patients are already maximally vasoconstricted due to cardiac pump failure, and further increasing afterload with vasopressors may be deleterious, particularly when targeting higher MAP goals [3]. In the distinct high-risk subgroup of patients with CA, which is commonly associated with abnormal cerebral blood flow autoregulation, retrospective evidence suggests that maintenance of a higher MAP may be considered to improve cerebral perfusion and neurological outcomes [6]. However, randomized controlled trials of higher MAP targets (80 or 85 to 100 mmHg) have not shown an improvement in neurological outcomes when compared to a target of 65 mmHg [18, 19]. Likewise, we did not observe a different MAP threshold for patients with CA and CS in our study. The use of higher vasopressor doses to achieve a higher target MAP after CA poses a risk of increasing the arrhythmia burden, which may be particularly harmful in CA patients with an arrhythmic substrate. As a result, current society guidelines for patients with septic shock or CA recommend MAP targets of 65–70 mmHg [6, 20].

It is crucial to note that the observed association between outcomes and mMAP_24_ demonstrated in our retrospective observational study is not the same as testing specific MAP goals for titrating vasopressor therapy in CS patients. We could not determine the MAP goals used by the treatment team, and therefore, we could not distinguish patients who had low MAP due to failure to achieve a prescribed MAP goal from those in whom a lower MAP was successfully targeted. Besides, patients with lower MAP had more severe illness by all relevant metrics and did not receive as many supportive cardiovascular procedures; we could not exclude the possibility that these patient-specific factors drove the adverse outcomes as opposed to the lower MAP itself. Importantly, CS patients may preferentially benefit from tailored vasopressor and inotropic support guided by hemodynamic data, such as those derived from a pulmonary artery catheter, rather than a “one-size-fits-all” approach [3]. Nonetheless, our data clearly show that an inability to maintain MAP ≥ 65 mmHg during the first 24 h after CICU admission is associated with adverse outcomes in CICU patients with CS.

### Limitations

This retrospective cohort study has a number of inherent limitations, including the potential for unmeasured confounders and missing data to have influenced the results. This single-center cohort may not fully represent the general patient population with CS. The mMAP_24_ values included both invasive and noninvasive MAP measurements, but we could not determine which MAP measurements were made using each method, and mMAP_24_ potentially included a mixture of both. Admission diagnoses are based on ICD-9 coding and may underrepresent the number of patients with CS and associated comorbidities. The inclusion of a mixed CICU population without available hemodynamic or echocardiographic data implies that some patients may have had non-cardiogenic or mixed cardiogenic-septic shock states. MAP data is limited to the first 24 h of CICU admission, so this study cannot evaluate the association between patient outcomes and MAP beyond 24 h. For this reason, we specifically focused on organ failure occurring during the first 24 h of CICU admission and cannot comment on later development of organ failure. Detailed data regarding vasopressor doses over time could potentially provide additional indication of illness severity in the context of mMAP_24_; unfortunately, these data were not available. Patients with mMAP_24_ < 65 mmHg were less likely to undergo PCI; the reasons for this are likely multifactorial and largely dependent on clinical factors and contraindications (e.g., severe shock, renal injury, or concern for cerebral anoxia). Unfortunately, our retrospective dataset cannot account for these clinical decisions. In addition, we could not determine the incidence of relevant cardiovascular adverse events attributable to vasopressor and inotrope therapy. Due to lack of data availability, we could not account for patient-level variables before CICU admission, including specific diagnostic or therapeutic interventions which took place before CICU admission.

## Conclusions

There was an inverse correlation between mMAP in the first 24 h and hospital mortality among patients with CS admitted to the CICU. Patients with a MAP below 65 mmHg during the first 24 h after CICU admission had an increased risk of mortality. These findings provide indirect support for a MAP target of 65 mmHg for most CICU patients with CS. Further prospective research should evaluate which, if any, MAP goals are optimal for patients with specific hemodynamic or etiologic subtypes of CS.

## Supplementary information

**Additional file 1:** **Supplemental Figure 1.** Patient flow diagram describing inclusion/exclusion criteria and patient groups. **Supplemental Figure 2AB.** Hospital mortality as a function of the 24-hour average mean arterial pressure (mMAP_24_), among patients with acute coronary syndrome (A) or heart failure (B). **Supplemental Figure 3AB.** Hospital mortality and incidence of severe acute kidney injury (AKI) as a function of the 24-hour average mean arterial pressure (mMAP_24_), among patients with (A) and without (B) cardiac arrest. **Supplemental Figure 4AB.** Hospital mortality and incidence of severe acute kidney injury (AKI) as a function of the 24-hour average mean arterial pressure (mMAP_24_), among patients with (A) and without (B) a pre-admission diagnosis of hypertension. **Supplemental Figure 5**. Hospital mortality as a function of the 24-hour average mean arterial pressure (mMAP_24_), among patients age 65 and older. **Supplemental Figure 6**. Mean values of systolic (circles), diastolic (arrows), and mean (diamonds) blood pressure over the first 1, 6, and 24 hours of the CICU stay.

## Data Availability

The dataset supporting the conclusions of this article is included within the article and its supplementary materials.

## Abbreviations

ACS: Acute coronary syndrome
AKI: Acute kidney injury
CA: Cardiac arrest
CICU: Cardiac intensive care unit
CS: Cardiogenic shock
HF: Heart failure
MAP: Mean arterial pressure
mMAP_24_: Average mean arterial pressure during first 24 h of CICU admission
