# Supplementary material for: Association between mean arterial pressure during the first 24 hours and hospital mortality in patients with cardiogenic shock
Source: Crit Care. 2020 Aug 20;24:513. doi: 10.1186/s13054-020-03217-6 (PMC7439249; doi:10.1186/s13054-020-03217-6)
Supplement: Supplementary file 1 — Additional file 1: Supplemental Figure 1. Patient flow diagram describing inclusion/exclusion criteria and patient groups. Supplemental Figure 2AB. Hospital mortality as a function of the 24-hour average mean arterial pressure (mMAP24), among patients with acute coronary syndrome (A) or heart failure (B). Supplemental Figure 3AB. Hospital mortality and incidence of severe acute kidney injury (AKI) as a function of the 24-hour average mean arterial pressure (mMAP24), among patients with (A) and without (B) cardiac arrest. Supplemental Figure 4AB. Hospital mortality and incidence of severe acute kidney injury (AKI) as a function of the 24-hour average mean arterial pressure (mMAP24), among patients with (A) and without (B) a pre-admission diagnosis of hypertension. Supplemental Figure 5. Hospital mortality as a function of the 24-hour average mean arterial pressure (mMAP24), among patients age 65 and older. Supplemental Figure 6. Mean values of systolic (circles), diastolic (arrows), and mean (diamonds) blood pressure over the first 1, 6, and 24 hours of the CICU stay. [file 13054_2020_3217_MOESM1_ESM.docx]

**Supplemental Figure 1:** Patient flow diagram describing inclusion/exclusion criteria and patient groups


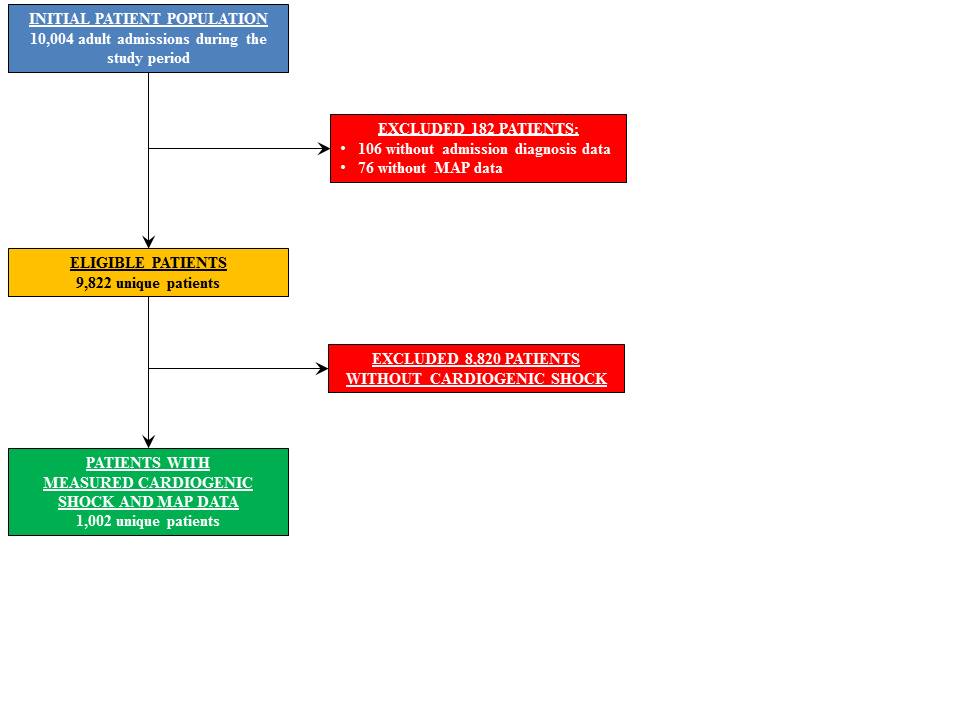


**Supplemental Figure 2AB**: Hospital mortality as a function of the 24-hour average mean arterial pressure (mMAP_24_), among patients with acute coronary syndrome (A) or heart failure (B).

**Supplemental Figure 3AB:** Hospital mortality and incidence of severe acute kidney injury (AKI) as a function of the 24-hour average mean arterial pressure (mMAP_24_), among patients with (A) and without (B) cardiac arrest.

**Supplemental Figure 4AB:** Hospital mortality and incidence of severe acute kidney injury (AKI) as a function of the 24-hour average mean arterial pressure (mMAP_24_), among patients with (A) and without (B) a pre-admission diagnosis of hypertension.

**Supplemental Figure 5**: Hospital mortality as a function of the 24-hour average mean arterial pressure (mMAP_24_), among patients age 65 and older.

**Supplemental Figure 6**: Mean values of systolic (circles), diastolic (arrows), and mean (diamonds) blood pressure over the first 1, 6, and 24 hours of the CICU stay.

**
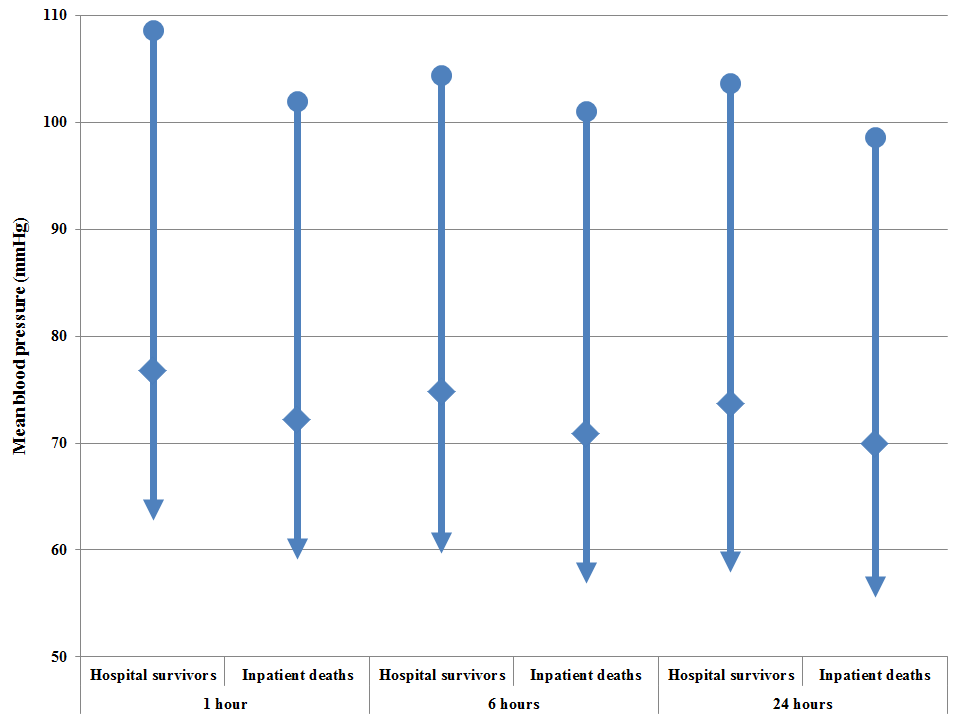
**
